# Development of MyTeen Text Messaging Program to Support Parents of Adolescents: Qualitative Study

**DOI:** 10.2196/15664

**Published:** 2019-11-20

**Authors:** Joanna Ting Wai Chu, Angela Wadham, Yannan Jiang, Robyn Whittaker, Karolina Stasiak, Matthew Shepherd, Christopher Bullen

**Affiliations:** 1 National Institute for Health Innovation, University of Auckland Auckland New Zealand; 2 Department of Psychological Medicine, Faculty of Medical and Health Sciences, University of Auckland Auckland New Zealand; 3 School of Psychology, Massey University Auckland New Zealand

**Keywords:** programs, mHealth, adolescents, parents, text messaging

## Abstract

**Background:**

Parents play an important role in the lives of adolescents, and supporting and addressing the needs of families continue to be the focus of many researchers and policy makers. Mobile health interventions have great potential for supporting parents at a population level because of their broad reach and convenience. However, limited evidence exists for such interventions for parents of adolescents. This study reports on the formative work conducted with parents and/or primary caregivers to identify their needs and preferences for the development of MyTeen—an SMS text messaging program on promoting parental competence and mental health literacy for parents of adolescents (aged 10-15 years).

**Objective:**

The aim of this qualitative study was to explore parents and/or primary caregivers’ perspectives around youth well-being, parenting, and parenting support and their input on the development of MyTeen SMS text messaging parenting intervention.

**Methods:**

A total of 5 focus groups (n=45) were conducted with parents or primary caregivers of adolescents aged 10 to 15 years between October and December 2017 in New Zealand. A semistructured interview guideline and prompts were used. Data were audiotaped, transcribed, and analyzed using inductive thematic analysis.

**Results:**

Participants were concerned about youth mental health (ie, stigma and increasing demand on adolescents), and a number of parenting challenges (ie, social expectations, time, impact of technology, changes in family communication pattern, and recognizing and talking about mental health issues) were noted. Importantly, participants reported the lack of services and support available for families, and many were not aware of services for parents themselves. A number of recommendations were given on the style, content, and frequency of developing the text messaging program.

**Conclusions:**

Findings from this qualitative work informed the development of MyTeen, an SMS text messaging program designed to increase parental competence and improve mental health literacy for parents of adolescents.

## Introduction

Depressive disorder is a major health issue among adolescents [1]. In New Zealand, prevalence rates are around 4% to 8% at 15 years, increasing to 17% to 18% by 18 years [2]. The serious developmental consequences of adolescent depression and associated treatment challenges once problems have developed underscore the need for programs aimed at prevention [3]. Parents play a significant role in shaping processes associated with adolescent well-being [4,5]. Relatedly, parenting programs aimed at strengthening parenting skills and increasing knowledge on adolescent development have led to positive effects on parent-adolescent relationships, parent well-being, and adolescent well-being [6,7].

There have been increasing calls to adopt a public health approach to parenting support [8,9]. The aim of the approach is to enhance parenting practices, competence, and adjustment for all parents and thus produce multiple beneficial health and developmental outcomes for young people at the population level [9,10]. However, poor participation by parents stands as the greatest barrier to widespread effective implementation of such programs [9,11]. This implies that a large segment of the population is failing to receive the benefits of such programs [8]. Many parents of adolescents are left without the support and information on the normative changes and parenting issues of adolescence that they need. Studies conducted with a diverse population suggest that all parents report a desire to learn about parenthood and that many families would welcome additional support on the task of parenting [12-15].

Mobile health (mHealth) interventions have great potential for supporting parents at a population level because of their broad reach and convenience [16]. mHealth offers a wide range of potential benefits over traditional approaches such as programs that can be delivered anywhere at any time; programs are more proactive (initiated by the service) than traditional services; they are flexible and can be personalized and tailored to specific cultural, age group, and health needs; reach is increased because the barriers of face-to-face contact (such as time, cost, and travel) are removed; and disparities in access across the socioeconomic status gradient are decreased because of the high penetration of mobile phones across these groups [13]. Specifically, mobile-based messaging programs may be particularly useful because of their simplicity and minimal burden for participants. Evidence suggest that SMS text messaging programs are effective in promoting parenting behavior change for parents of young children (eg, decreasing the likelihood of abuse and neglect, increasing childhood vaccinations, and encouraging healthy pregnancies) [17-21]. The interventions were well received by parents of various population, including those that are socially deprived [18,21].

Although there is evidence for SMS text messaging programs for parents with young children, its application among adolescent parent populations are unknown. There is considerable potential in New Zealand to supplement and enhance existing services for families by leveraging mHealth technologies in delivering programs for parents. A total of 92% of New Zealand households have access to a mobile phone with no differences in internet access or mobile phone ownership by ethnicity or education and few differences by age below 65 years. As such, we developed a program of text messages adapted from the Parenting Strategies Program, a set of evidence-based parenting guidelines developed through (1) a systematic review and meta-analysis of parental factors associated with adolescent depression and/or anxiety and (2) a Delphi study of international expert consensus about actionable strategies parents can use to reduce their child’s risk of depression and anxiety. The guideline is Web-based and freely available [22]. Of the 13 domains, 9 (helping your child to deal with problems, helping your child to deal with anxiety, minimizing conflict with your child, encouraging supportive relationships, encouraging good health habits, supporting your child’s autonomy, avoiding criticizing your child, establishing family rules and consequences, and improving your relationship with your child) were used to guide the development of the text messages. These domains were chosen as it has been found to be particularly useful for parents of adolescents. Key information from each domain were edited to a maximum of 160 characters (maximum length of text). Moreover, 1 or 2 sample text messages were derived from each domain.

We report on formative research conducted with parents of teenagers (aged 10-15 years) to seek their perspectives around parenting adolescents and parenting support. Specifically, we were interested in parents’ views of their needs in supporting their adolescent’s well-being, the extent to which they were aware of support and services available to them, and their input for the development of a mobile-based SMS text messaging parenting intervention.

## Methods

### Overview

This study is part of a wider project that aims to develop and evaluate the effectiveness of MyTeen, an SMS text messaging intervention program that promotes parental competence and mental health literacy for parents of adolescents. The protocol of the randomized controlled trial is published elsewhere [23]. To inform the development of the SMS text messaging program, 5 focus groups (n=45) were conducted from October to December 2017.

### Recruitment

Efforts (ie, recruitment via youth rugby sports club, cultural organizations, word of mouth via personal networks, and individuals were also encouraged to forward information about the intervention to others who might be interested) were made to recruit a diverse sample, including Mãori (indigenous people of New Zealand) and Pacific parents, who are considered as hard to reach. To maximize recruitment effort, 1 of the research assistant who identified as Mãori actively engaged with ethnic minorities via her own network and promoted the visibility of the study within the Mãori community. Promotion materials directed interested participants to contact a member of the project team to find out more about participation. Eligibility for the study was ascertained at that time. Individuals were eligible to participate if they were a parent/primary caregiver of a teenager aged 10 to 15 years, able to speak English, and able to attend a focus group. Eligible participants then received information about the study, and those who agreed to participate were offered a time and place to join a focus group.

### Ethics Approval

Ethics approval was obtained from the University of Auckland Human Participant Ethics Committee (UAHPEC, Ref 019659). Participation was voluntary. Written consent was collected, and a questionnaire on sociodemographic characteristics was completed before the start of the focus group.

### Data Collection

Each focus group lasted between 60 and 90 min and was conducted by 2 trained moderators experienced in conducting focus groups. Group size ranged from 3 to 15 participants. Sessions were audio-recorded with the consent of the participants. At the beginning of each focus group, participants were given information on the overall aim of the study including the development and trialing of the SMS text messaging program. A topic guide and prompts were developed based on a standardized questioning technique to cover a range of key issues related to the research questions. A total of 5 questions were asked: what are your perceptions about mental health and well-being among youth?, what are your perceptions on parenting teens?, how accessible is mental health support at school or in the local community?, what resources or support would you like to see for youth and families in your community?, and what are your thoughts around an SMS-based mobile intervention for supporting parents?. In addition, a list of sample text messages from the SMS text messaging program was shown to parents to comment and provide feedback. The focus groups were conducted with flexibility to allow unanticipated themes to emerge. Refreshments were provided, and each participant received a NZD $50 voucher in appreciation of his or her time and transportation cost.

### Data Analysis

Transcripts were imported into NVivo (Version 11.0; QSR International) to allow for electronic coding and retrieval of data. The data were analyzed using thematic analysis [24]. This method was deemed appropriate for summarizing the data and identifying patterns in the data to provide an interpretation. The 6 steps outlined by Braun and Clarke [24] were followed. The first author and an experienced research assistant familiarized themselves with the data by thoroughly reading, rereading, and annotating the material with preliminary ideas. Initial themes were generated based on inferences from the data. The data were then categorized into these themes. Subthemes were developed to more clearly capture the data.

As a mean of assessing credibility of the coded themes and to minimize bias, 2 researchers performed the coding analyses. A small number of differences were found and were resolved through discussion. A third researcher reviewed the transcripts to further ensure that the themes identified reflected the data. Field notes were also constantly referred to as supplementary materials. Peer debriefing was also used and involved ongoing discussion between researchers during the course of focus groups. Finally, informal member checking occurred during data collection when the moderator reflected back on her understanding of participant responses and sought feedback regarding whether this understanding was accurate.

## Results

### Participants

Of the 45 participants, 27 were mothers, 7 were fathers, 3 were grandparents, and 8 identified as others (siblings and relatives). Over half (24/45, 53%) of the participants were identified as married, 29 (13/45) as single, and 18% (8/45) as divorced. Participants were mostly Mãori (3/45, 62%), 16% (7/45) were European, 13% were Pacific (6/45), and 9% (4/45) identified as others. Majority (32/45, 71%) of the participants reported completing at least high school education. Several themes emerged from the focus groups and are reported below.

### Themes

#### Concerns Around Youth Mental Health

Participants were concerned about the high rates of youth depression and suicide in New Zealand. Sadly, many of our participants knew of a young person (directly or indirectly) that had either experienced depression, self-harm, or suicide:

When you have a look at the subject (suicide) itself, talk about mental health, there wouldn’t be anyone that hasn’t been touched. It’s just something that, you know, the stats back it up. It’s terrible.

#### Stigma Around Youth Mental Health

The stigma attached to youth mental health was a prominent theme. Participants discussed the terminology itself as problematic and come with a negative connotation:

When you look at the way kids use the word mental, you know, it’s someone that’s done something really stupid, you’re mental, you’re deranged or whatever. It’s got a stigmatism that comes with it.

Participants also reported having experienced stigma associated with asking for help as it was seen as a sign of weakness or an indication that their family was not coping. Participants acknowledged that for a very long time, people did not talk openly about mental health issues and for a number of reasons such as concerns over being judged or treated differently, burden to the family, and denial:

I think the biggest problem is that people are afraid to admit it.

#### Increasing Demands on Adolescents

Participants felt that many of today’s young people are experiencing high levels of stress, stemming from high expectations for academic achievements, jobs, social media, and peer pressure:

Just seems to me the kids are under a lot of stress, a lot of pressure, social media stuff, bullying, social media bullying. Peer pressure.

I think there’s a lot of pressures these days, more for academic success than in our days. The pressure to have a job and stuff as well. I mean its money, it’s everything. The society today is just one big pressure for them all.

#### Increasing Challenges for Parents

Parents expressed that at times they are overwhelmed by the expectations and social pressure to be a “good parent” and raise competent adolescents. Time and juggling multiple demands throughout the day were frequently mentioned as a parenting challenge:

I think society’s expectations on what your child should be and what it shouldn’t be and that’s really hard to juggle and challenge.

Most parents I think are guilty of this, is making sure that I actually allocate proper quality time but work and life takes over and you get so caught up in everything else.

Technology contributed to the challenges parents experienced, which were not present in previous generations. For example, parents spoke about cyberbullying, problematic screen use, and adolescent access to internet content. Parents noted that these issues all had an impact on adolescent well-being:

Social media has a huge impact (on their wellbeing).

They can just Google any word. It will bring up porn, it will bring up conspiracy theories, it will bring up anything and it just impacts their view straight away.

#### Changes in Family Communication Pattern

All participants agreed that family communication pattern has changed significantly. Participants felt that the increased use of technology devices created a communication barrier within the family. The quality of communication has also changed, with adolescents using text or social media instead of face-to-face conversations with parents:

I find it frustrating that they, they just can’t communicate anymore, like they can’t speak to a person. It’s all done with phones or messages, no one seems to be able to pick up a phone or confront somebody directly.

I had my granddaughter around home with her friends, and they are all sitting there, head down. And then suddenly they’ll all laugh at the same time. What was the point of you all being in here?

#### Challenge With Recognizing and Talking About Mental Health

A number of our participants noted that they did not know how to best respond to and assist their adolescents if they were experiencing problems. For some, the challenge was recognizing the symptoms:

The thing for me is I know a few people that have committed suicide, and the people that have been in contact with them that day, or even the week leading up to it, they seem to be the happiest they’ve been in a long time. They often set a date that they’re going to do it, and they’re happy because they’re going to be out of whatever misery. If the signs were obvious then you will be able to tackle it, but it’s not.

Participants felt that it was difficult to talk with their adolescents on mental health issues and expressed their fears about what and how much to say. Others felt uncertain or incompetent about what information to give and where to direct their teens for support:

That’s what I said to my wife, coz we said we need to talk about this, and the first thing I said was does she even know what suicide is, or what that means when a person does that? But we had to, you know, and that was my fear, are we giving them information of an option, which was a fear.

Participants also noted that their adolescents did not want to talk to them about mental health issues:

Even once you notice that something is up with your child, the challenge then is to open that channel of communication with them. You do get that (from them), “No, I am alright, there’s nothing wrong, I am okay.”

They’ll tell you there’s nothing wrong with them, and yet what I’ve found, my moko (grandchild) committed suicide the other day. She was 10, these are things that we should know about.

#### Lack of Mental Health Support From Schools

When asked about the level of support in schools and in their community, many of our participants were dissatisfied. In particular, concerns were expressed about the quality of school-based counseling services. All participants agreed that the resources at schools were insufficient, including the student-counselor ratio, lack of cultural sensitivity, poor parent-teacher communication, and the barriers associated with accessing school-based services. In New Zealand, school deciles are a measure of the socioeconomic position of a school’s student community relative to other schools throughout the country. Lower decile rating indicates higher proportion of students from low socioeconomic communities:

My school are decile one and two so we get nothing.

My son, his school’s a decile nine and there’s not even a social worker or anything there.

One parent spoke about her experience and disappointment with the school:

She was going downhill in all of her mahi (work) and all of that. And the teacher gave me a leaving form, he didn’t offer any kind of support. This was the dean, so no, (school services) not the greatest.

Another parent described the concerns around confidentiality and the stigma associated with one’s reputation when seen walking into a counselor’s office at school:

I couldn’t take my daughter to the school because if they got called up to the counsellor at the school she uses a particular coloured piece of paper. So all the kids would know where you’re going, like they can tell by whatever slip they’re given. So there was no way she would speak to a counsellor through the school.

#### Lack of Mental Health Services for Adolescents

Apart from schools, parents were only able to name 1 or 2 services that they knew of for supporting youth. These included Child Youth Services, WINZ, and Youthline. A few participants who knew about it from personal experience were concerned that these services were only directed to those already in crisis and stressed the frustration in having to wait too long before a professional contact could be established. Only 2 of our participants knew about other supportive services (eg, Te Kawa te Rangatahi); these participants knew about this as they worked in social services:

I had a problem with my teenagers and what I did was I went to organisation after organisation until I got help from them. I just kept going and going until I got help.

When it comes to this age group, the teenager, there’s not many (services). There’s mental services but they are too young to get into a lot of the services. There’s Youthline, but yeah. So Dr Google, GP, and schools.

#### Lack of Services for Parents

Even less was known about services available to parents. Participants were unable to identify any formal parenting support services beyond their family doctor, Plunket, and Well Child Tamariki Ora, with the latter 2 being Ministry of Health services provided to New Zealand families and are directed at parents of young children (aged 0-5 years). Parents indicated that they do not know where to access information, ask questions, or seek advice or formal support when needed. Parents often turned to family, friends, and the internet for advice:

I’ll be honest, I wouldn’t know where to go if my kid had any issues. And I think a lot of it is just left up to us (as parents) these days, because the systems just aren’t there for us.

#### Recommendation for Youth and Parent Services

Overall, participants expressed a need for more accessible services for both adolescent and parents:

Get the information out there, just flood with information about feelings and emotions and the normality of them.

Education, we need to know more about everything, like you know teen emotions, mental health.

Participants noted that it was important for adolescents themselves to recognize the mental health issue and seek support when needed:

How are we to support them when they don’t even understand themselves what mental health is and what support? It’s really them that should be creating their own supports in schools and communities. But then again, there’s a lot of schools that wouldn’t allow it (suicide) to be talked about. So how are we supposed to educate our tamariki (children) if the schools can’t even educate them properly on mental health, and what that means.

Participants suggested several support services for adolescents. This ranged from technology-based programs (eg, apps, SMS text messaging, and Web-based) designed specifically for adolescents to incorporating mental health education within schools, increasing school counseling resources (eg, full-time dedicated trained staff that are culturally sensitive and after school drop in), and public mental health promotion campaigns. Nonetheless, many acknowledged the challenges with their suggestions (eg, lack of funding and resources):

Our (suicide) rate being very high, they should try and have better counselling/support services at all schools. Going to secondary school, there’s not much help. I think there should be a compulsory thing happening at secondary schools, because that’s when most of our teens are taking their lives.

The Education Board’s not going to pay too much for more social workers in the schools. It comes down to money in the end.

Parents also spoke about support that they wanted as parents. This includes easily accessible information; parenting programs that were brief and not time consuming, or that they can do at their own pace; and parenting apps.

#### Text Messaging Parenting Program

Following discussion on accessing and increasing support, participants were reminded about the wider project to develop and evaluate the SMS text messaging program that promotes parental competence and mental health literacy for parents of adolescents. Participants were given sample text messages to review, and their input on the style, content, and frequency of the intervention were sought. Overall, parents were enthusiastic about receiving parenting information via text messages. Participants noted that it was a valid approach to engagement, as mobile phone usage is widespread among older generations (eg, grandparents). They also thought that the text messages could be shared with other family members and serve as a catalyst to engage in conversations.

All participants agreed that the intervention would be best delivered in English, and other languages were optional. Participants were aware that the program was proposed to be brief and preventative; hence, personally tailored messages did not matter for many of our participants. They were also aware of the wording limit (160 characters) in text messages, and many of the preferred to receive simple, jargon-free, nonjudgemental, and direct information. A majority of participants mentioned that the text messages should be fun to read, including the use of emoji and images:

Keep it simple with the words. Don’t make them too long, coz then you switch off.

Use emoji, sometimes it’s easier to just send a heart, than to actually type I love you.

Provision of practical parenting tips and strategies, knowledge about adolescent development, symptoms of mental health problems, and where to access information were viewed as important content to include in the program. This was consistent across focus groups:

Where to find information, where to get help.

Brain development and stuff, if it’s normal, the behaviour that they’re doing because of their brain development.

Parents also wanted messages on self-care and acknowledgment of their parenting effort. One parent communicated the importance of positivity. This sentiment was echoed across focus groups, with participants frequently mentioning the need for encouraging messaging. Participants provided sample wordings such as “you’re not alone,” “you are doing a good job as a parent,” and “just breathe.” Many parents wanted the program to emphasize that they were not alone and support is available when needed:

Sometimes you just need to see a little ray of sunshine.

Supportive little notes for parents.

A small number of parents mentioned the importance of including spirituality and cultural relevance in the text messages:

Reminding us the connection with the land, with our forest, or with our sea. Brings you so much peace.

There was no general consensus about how often the text messages should be delivered and for how long. Some participants preferred a daily text message, whereas others thought 2 to 3 messages per week was sufficient. Similarly, some participants thought the text messages should be ongoing beyond the intended 1 month; others felt that the duration was sufficient or that it depended on the individual receiving the program.

## Discussion

### Principal Findings

In this study, we examined parents’ perspectives on youth well-being, parenting, and parenting support and their input on the development of MyTeen text messaging parenting program. There are several findings from our focus groups that are particularly noteworthy.

The need to support parents of adolescents was apparent. Many of the challenges (eg, social pressure to be a “good parent,” lack of time, the impact of technology, and parent-adolescent communication) reported are universal and consistent with other studies [25-27]. New technology, in particular, pushes boundaries of parenting and creates new challenges for today’s parents [28,29]. For example, the internet creates informational freedom and changes the nature of social interactions among individuals [30]. Several studies have reported negative association between internet use and the quality of parent-adolescent relationships. Although outside the scope of our study, more research is needed to examine parenting with respect to adolescents’ use of technology and its impact on family dynamics. For example, a child’s greater familiarity and proficiency with technology could undermine parental authority and potentially adversely affect parenting and parent-child relationships [31].

It was evident that many of our participants lacked competence on initiating discussion about mental health with their child. Parents are one of the most influential sources by which adolescents learn to label, identify, and interpret emotions. Parents also act as an important change agent in assisting young people to access help when needed [32]. Equipping parents with knowledge and tools to help them foster emotional competence and identify mental health problems in young people, raising awareness and understanding of the professional help that is available, is particularly salient in preventing and mitigating adolescent mental health problems. Increasing parents’ self-efficacy can further play a role in buffering their adolescents from mental health problems [33-35].

There is a lack of available services for parents and adolescents. Barriers related to not knowing where or how to seek help was particularly salient, suggesting the need for strategies to raise awareness of available services. Majority of services to support youth mental health focus on the adolescent themselves, with scant knowledge of, or attention to, the unique role and needs of their parents [36]. Parenting interventions can promote parents’ self-efficacy and mental well-being and improve knowledge and skills to support their adolescent well-being [6].

Parents had many suggestions for services directed at teenagers, including technology-based programs (eg, apps, SMS text messaging, and Web-based), mental health education within schools, school counseling resources, and public mental health promotion campaigns. Importantly, the idea of an SMS text messaging program to support them as parents was well received. Time was constantly stated as a barrier to accessing parenting support; hence, text messages that are proactive (initiated by the service) and do not require attendance by the participant were appealing for our participants. Although SMS text messaging may not be considered a “novel” mobile phone app it remains the most widely used [37]. Parents offered suggestions for the SMS text messaging program including the message tone, content and length, as well as the frequency of text messages delivery. As noted previously, parents perceived challenges with adolescent use of technology; however, technology has become an integral part of everyday life, including the parents themselves. Much more research is needed to understand how we can balance the benefits and negative impact of technology in families.

On the basis of the qualitative findings, we refined the sample text messages derived from the Parenting Strategies Program and developed additional text messages based on parents’ needs around self-care and providing resources for help seeking when needed. Some of the domains (eg, helping child to deal with anxiety, avoid criticizing your child, and establish family rules) were omitted as (1) it appeared to be of lesser importance to parents than other domains and (2) the intervention was short in duration; hence, not all domains can be included. Positive tone, emoji, and jargon-free wordings were all taken into consideration when designing our program. Sample text messages from MyTeen are shown in Table 1.

**Table 1:** Sample text messages from MyTeen.

Domains	Sample text message
Establish and maintain a good relationship with your teenager	Show your teen love and affection, talk with them and have fun with them! By doing so you’re making a huge contribution to their emotional wellbeing.
Be involved and support increasing autonomy	Spend quality time together. Don’t get caught up in to-do lists & screens. Have a meal, take a walk, play a game, or make a list of things to do together.
Minimize conflict in the home	Minimise conflict in the home and remember you are the role model for your teen! Stay calm & encourage them to find solutions to problems/conflict.
Encourage good health habits	Sleep is really important for your teen! Encourage them to switch off/reduce the time they spend on their phone or devices a few hours before bedtime.
Knowledge and risk of depression	Depression = feelings of sadness & irritability lasting longer than 2 weeks, affect daily life and stop people from taking part in things they used to enjoy.
Encourage professional help seeking when needed	If u need some help or support, don’t hesitate to ask. Local GP, Parent Helpline: 0800 568 856, Family Services: 0800 211 211, http://www.commonground.org.nz
Parental self-care	Take care of yourself!!! Take a break, and get support for yourself too. If your own needs are met, its easier to be patient, consistent & available to your teen.

### Limitations

There are limitations in our study. The overall number of participants was small and not intended to be representative of the wider parent population. Nonetheless, we were successful in recruiting a high proportion of hard-to-reach parents (ie, Mãori and Pacific parents). Moreover, many ideas and themes were repeated as the focus groups progressed, indicating that saturation might have been reached.

The decision for developing an SMS text messaging program (rather than an app/Web-based program) was made before conducting the focus group and thus may have influenced the discussion on parenting support. Nonetheless, our participants considered text messages as feasible and were keen to try the program when available.

### Conclusions

Despite the limitations, our study reinstates the need for accessible and family-friendly parenting outreach and services. Wider access to advice and information is now feasible by utilizing technology such as mobile phone and Web-based platforms [16]. The widespread use of mobile phone suggests that parenting programs can incorporate mHealth technology with minimal economic burden. Although a brief SMS text messaging program may not meet the need of families requiring more intensive intervention or replace face-to-face therapy, it may offer support for prevention and early identification of emerging issues to many more parents who otherwise would have had none. Findings from this qualitative work informed the development of MyTeen, an SMS text messaging program designed to increase parental competence and improve mental health literacy for parents of adolescents [23]. Future studies may wish to examine the perspectives of adolescents themselves and how family can be better supported. The effectiveness of this program has been evaluated in a randomized controlled trial, and the main results will be reported elsewhere.

## Abbreviations

mHealth: mobile health
